# Alleviation of D-gal-induced senile liver injury by Rg3, a signature component of red ginseng

**DOI:** 10.18632/aging.204819

**Published:** 2023-06-21

**Authors:** Ke Xu, Biwen Hu, Xuhui Ding, Zhengyu Zhan

**Affiliations:** 1 Department of Clinical Medicine, Medical College of Nanchang University, Nanchang 330000, Jiangxi Province, P.R. China; 2 Department of Oncology, The First Affiliated Hospital of Nanchang University, Nanchang 330006, Jiangxi Province, P.R. China; 3 Department of Surgery, The Second Affiliated Hospital of Jiaxing University, Jiaxing 314001, Zhejiang Province, P.R. China

**Keywords:** Rg3, red ginseng, D-gal, liver injury

## Abstract

To investigate the mechanism by which ginsenoside Rg3 regulates oxidative stress (OS) and inflammation through NF/KB pathway to delay mouse liver injury.

This work randomized Balbc mice as four groups: Normal, D-gal, Rg3-L, Rg3-H. Paraffin-embedded liver tissue sections were prepared, later, BAX/BCL-2 protein expression was observed by HE, Sirius red, TUNEL and immunofluorescence to detect apoptotic injury and α-SMA/TGF-β protein expression to detect fibrosis, and liver inflammation-related protein NF-KB was detected.

HE and TUNEL staining showed that Rg3 reduced necrotic cells and fibrosis in liver-injured mice, Rg3 increased anti-inflammatory cytokine IL-18 and reduced TNF-α, IL-1β and IL-6 expression. Conclusion: Ginsenoside Rg3 can effectively antagonize D-gal’s role in mouse liver injury, and its mechanism may be associated with regulating inflammatory pathway by Rg.

## INTRODUCTION

As estimated by statics from the Global Burden of Disease, there were over 2 million individuals dying from hepatopathy in 2010, mostly consisting of acute hepatitis, liver cancer and cirrhosis, occupying approximately 4% of all death cases worldwide [1]. As a body organ with critical role in metabolism, liver injury may result from a variety of factors, such as drug toxicity, hepatitis viral infection, bacterial metabolites, industrial chemicals or drinking [2]. Liver apoptosis and chronic fibrosis are important stages in the development of liver disease [3]. How to reverse liver damage during this stage is an important tool in preventing liver disease. Continuous injection of D-gal induces excessive oxidative free radical generation in the body and is a classical model of near-natural ageing [4], which is why it has been extensively utilized for exploring aging mechanisms and anti-ageing drugs [5]. The global population with the age of more than 65 years may elevate from 524 million to approximately 1.5 billion during 2020-2050. Older people are particularly vulnerable to chronic diseases, especially damage to organs [6].

Ageing-induced liver damage is mainly characterized by excessive inflammation and dysregulation of enzymatic activity in the liver. Regulation of the conversion of hepatic stellate cells to myofibroblasts is essential to prevent senescence-induced liver fibrosis [7]. Liver fibrosis is accompanied by lipid deposition and overproduction of alpha-smooth muscle protein (α-SMA). Due to excessive inflammatory factor expression, inflammatory factors infiltrate the liver tissue and induce excess transforming growth factor-beta (TGF-β) generation [8], which induces the activation of myofibroblasts. In addition to this, dysregulated levels of oxidation in the liver further induce liver damage. Among other things, ROS are transmitted to mitochondria via the nicotinamide adenine dinucleotide phosphate (NADPH) oxidase system as well as the respiratory chain [9, 10]. Apart from mitochondria, overexpression of cytochrome P450 2E1 (CYP2E1) and 4-Hydroxynonenal (4-HNE) further damage hepatocytes [11].

In addition, a number of natural components or products are analyzed in clinical studies because of the possible medicinal effects. Ginseng is the root of the natural medicinal plant ginseng. Ginseng has long been used as an ethnic medicine in Asia for over 2000 years [12, 13]. Interestingly, processed ginseng, red ginseng, has better antioxidant activity. Studies have reported that red ginseng has been widely used for a variety of conditions such as neurodegeneration, gastroenteritis, liver damage, and aging [14]. Chemical composition studies have shown that the Rg3 content in red ginseng is 8.6 times higher relative to that in regular ginseng. Due to the good antioxidant and anti-inflammatory activities of Rg3, it is hypothesized that it can mitigate the ageing process of the liver by resisting hepatic oxidative stress (OS).

## MATERIALS AND METHODS

### Materials

This study acquired 20 (R)-G-Rg3 (purity≥98.0%, HPLC) and evaluated the quality according to our prior description. In addition, the present work obtained D-gal from Sigma-Aldrich Chemical Co. (St. Louis, MO, USA), whereas BCA protein assay kit (P0010) and RIPA lysis buffer (P0013B) in Beyotime Biotechnology Co., Ltd. (Jiangsu, China). Additionally, we purchased alanine aminotransferase (ALT), aspartate aminotransferase (AST), total cholesterol (TC), triglyceride (TG), high/low density lipoprotein cholesterol (LDL-H/LDL-C), and hematoxylin-eosin (H&E) kits in Nanjing Jiancheng Institute of Biological Engineering (Nanjing, China). The present work acquired Mouse IL-1β, Mouse IL-6, Mouse IL-18, Mouse TNF-α ELISA kits in Multi Science Biotechnology Co., Ltd. (Hangzhou, China).

### Animal and experiments

The present work obtained the 8-week-old ICR male mice weighing 24-26 g) from Changchun ESI Laboratory Animal Technology Co., Ltd (SCXK(JI)-2020-0001, China) and raised them within the pathogen-free rearing room under the 60.0% ± 10.0% humidity, 22.0° C±2.0° C and light/dark cycle of 12-h/12-h. The animals were allowed to eat food and drink water freely. At 1-week following domestication, animals were randomized as 4 groups (n=6): Normal, D-gal, D-gal+Rg3 (25 mg/kg) group and D-gal+Rg3 (50 mg/kg). For the remaining mice excluding the Normal group, intraperitoneal injection of D-gal (600 mg/kg) was given for two weeks consecutively for modeling liver senescence. Rg3-treated mice were given gavage from the fourth week of feeding.

**Figure 1:**
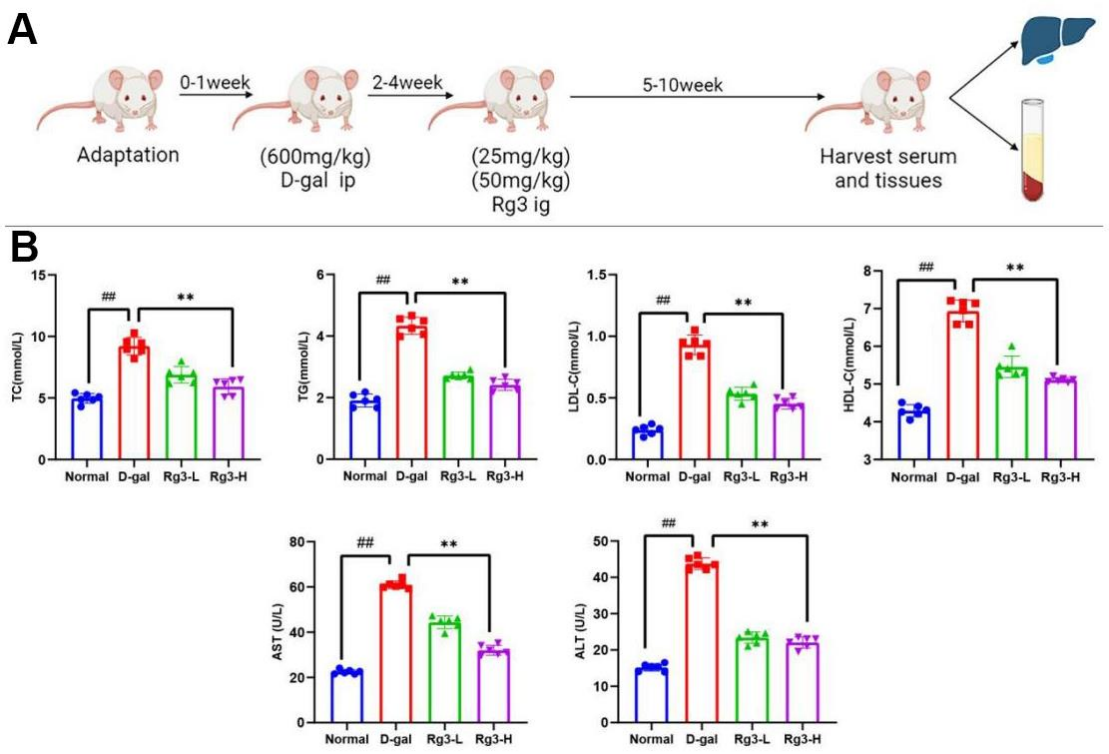
(**A**) Sketch map showing D-galactose mediated mouse aging and damage models. (**B**) Serum lipid metabolism and liver function indexes in mice. Results are represented by mean±S.D. #p< 0.05, ##p<0.01 vs. Normal group. *p<0.05, **p<0.01 vs. D-gal group.

This work calculated doses and determined treatment schedules according to our preliminary results together with additional prior findings. (Figure 1A).

### Evaluation of serum biochemical indexes

This study used ALT and AST kits to detect the main indexes of liver function [15]. Briefly, after mice were killed, blood sampling and centrifugation at 1000 × g and 4° C for a 10-min period were completed to collect serum. Later, the kit-derived specific substrate was added into the 96-well plates under 37° C for a 30-min period. Absorbance (OD) value was measured at 510 nm. Additionally, TC, TG, HDL-C and LDL-C levels in serum were also determined for analyzing liver lipid metabolism function [16, 17].

### Immunofluorescence (IF)

After deparaffinage, liver sections were subject to rehydration, immersion in 0.3%H2O2 for 5min to eliminate endogenous peroxidase, and 15-min incubation with citrate buffer (pH 6.0) to achieve antigen recovery by boiling. Later, 0.1%Triton X-100 was added to permeate sections, followed by treated with 10% goat serum for non-specific antigen blocking [18].

After washing with PBS, secondary antibodies (1:200) including Alexa Fluor 488-labeled goat anti-rat and 594-labeled goat anti-rabbit were added to further incubate sections. The samples were incubated for a 10-min period within the DAPI working solution under ambient temperature in the humid chamber in dark. The fluorescence microscope was employed to capture images. Signal intensities were analyzed using ImageJ software and determined through calculating mean unit intensity to control [18].

### TUNEL staining

Mouse livers were taken and embedded in paraffin, followed by cutting in 4-μm sections with the rotary microtome, dewaxing in xylene as well as dehydrated with ethanol. Liver apoptotic damage was assessed by the TUNEL Apoptosis detection kit in line with specific protocols, and DAPI staining was calculated to determine total nuclear number [19].

### Statistical analysis

SPSS18.0 software (IBM) was employed for statistical analysis. T test was utilized to analyze significant difference for two groups, while one-way ANOVA combined with Tukey’s test for three groups. Results were represented by mean±SD of 3 separate assays. P<0.05 stood for statistical significance [20, 21].

## RESULTS

### Rg3 alleviated abnormal liver function and lipid metabolism markers in mice

As shown in Figure 1B, TC, TG, HDL-C and LDL-C levels in serum of D-gal group remarkably increased (P<0.01). Their levels were decreased after Rg3 addition, which indicated that Rg3 could alleviate the abnormal liver lipid metabolism induced by aging.

ALT and AST represent major indicators of liver function. In this study, their contents of liver injury group remarkably elevated by D-gal injection (P<0.01), indicating the successful construction of mouse liver injury model. At 5-week post-Rg3 treatment (25 and 50 mg·kg−1), ALT and AST contents in serum of Rg3 treatment group decreased (P<0.01).

### Rg3 alleviates D-Gal-induced mouse liver apoptosis and fibrosis

**Figure 2:**
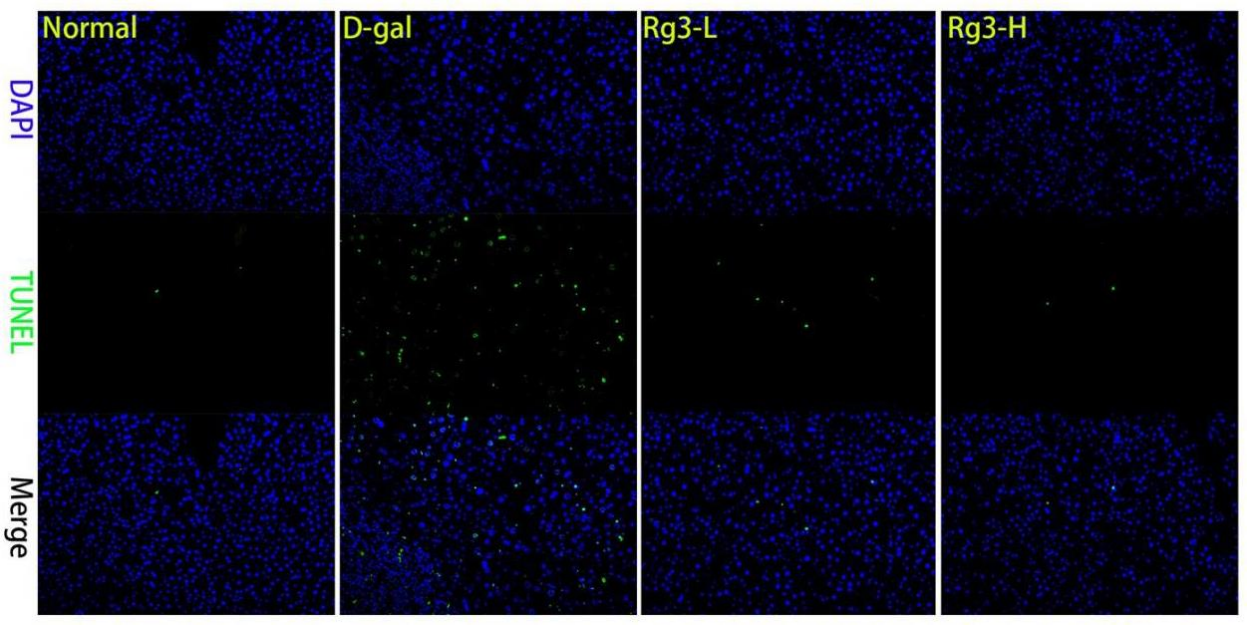
**Morphopathological section of mouse liver.** Mouse liver was stained by TUNEL section.

For investigating how Rg3 affected liver necrosis and fibrosis caused by aging, mice were intraperitoneally injected with D-gal to construct the liver aging mouse model, followed by Rg3 treatment. According to TUNEL staining, lobular destruction, apoptosis, collagen deposition and septal generation of D-gal group significantly elevated relative to Normal group, which were significantly reduced after Rg3 administration (Figure 2).

### Rg3 ameliorates the inflammatory response to D-GAL mediated liver injury through reducing serum cytokine accumulation in mice

**Figure 3:**
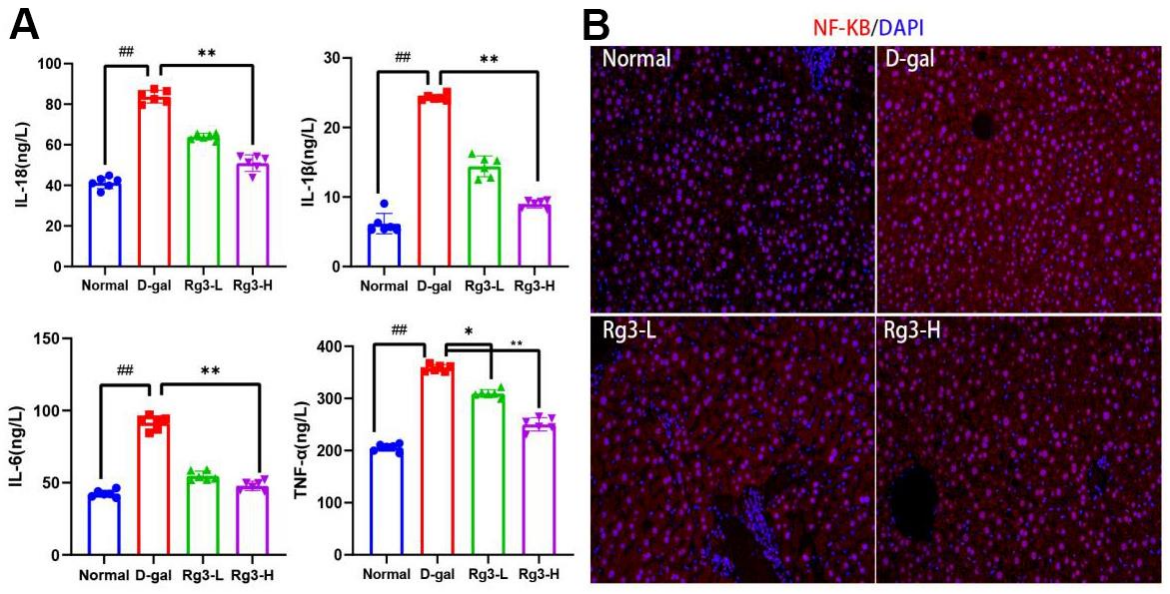
**Expression levels of inflammatory related factors induced by Rg3 on D-gal in mice.** (**A**) Serum inflammatory cytokines in mice. (**B**) Immunofluorescence section of NF-KB in mouse liver. Results are represented by mean±S.D. #p<0.05, ##p<0.01 vs. Normal group. *p<0.05, **p<0.01 vs. D-gal group.

Chronic inflammation in the body represents one of typical aging manifestations. Serum IL-18, IL-6, IL-1β and TNF-α contents were determined (Figure 3A). Compared with Normal group, D-gal exposure dramatically elevated IL-18, IL-6, IL-1β and TNF-α contents, and these changes were reversed after Rg3 treatment (p<0.01). For direct demonstration of the role of D-gal in inducing mouse liver inflammation, NF-κB protein expression was analyzed through IF staining (Figure 3B). NF-κB is a key protein regulating inflammation switch, and its expression level is closely related to cytokine secretion. The results showed that Rg3 antagonized the NF-κB overexpression, which displayed cytoplasmic and nuclear localization within D-Gal-mediated liver tissues.

### Rg3 plays an antifibrotic role by inhibiting D-Gal-mediated apoptosis

**Figure 4:**
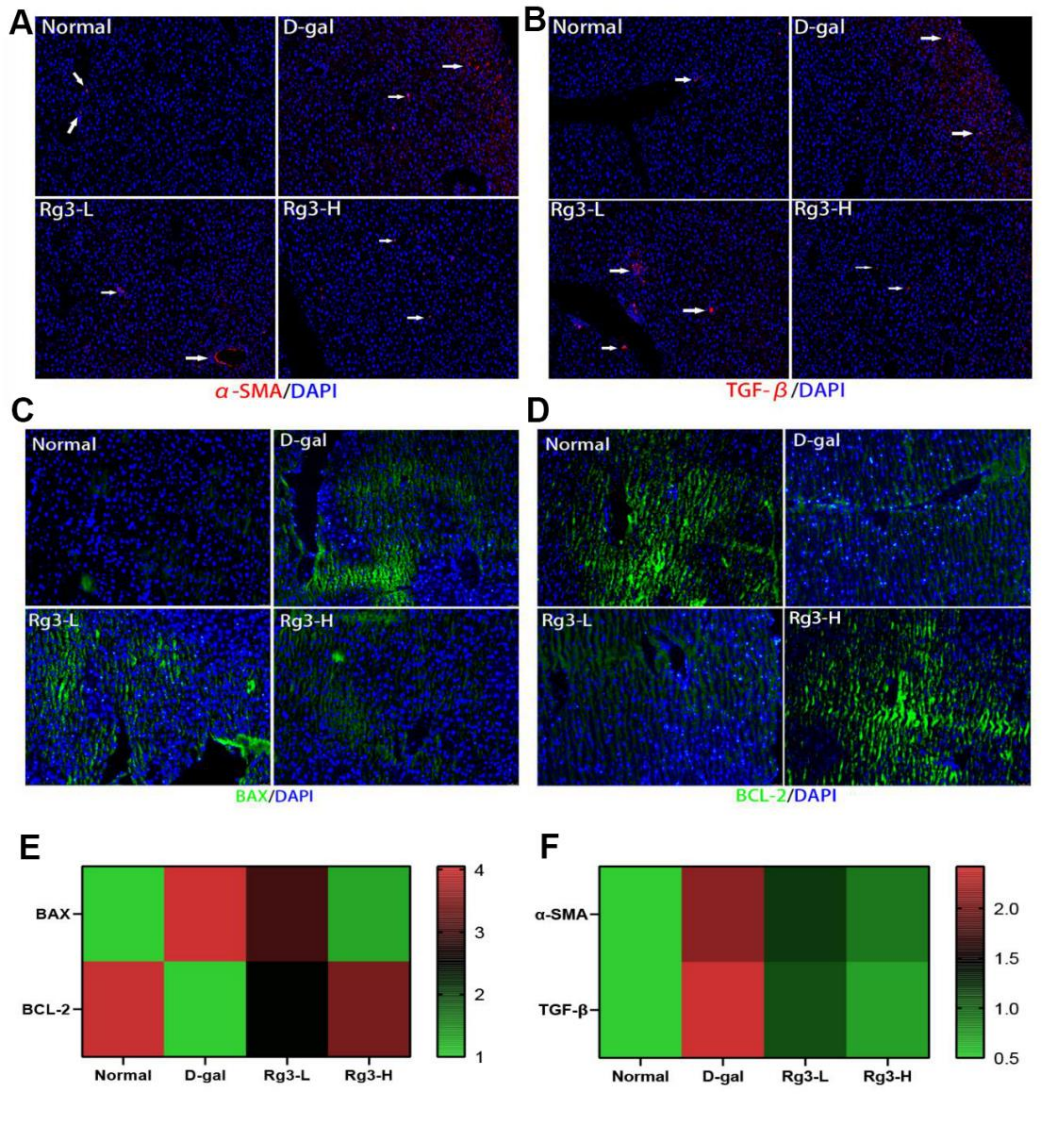
**Immunofluorescence section of mouse liver fibrosis and apoptosis-related factors.** (**A**) Immunofluorescence section of mouse liver with α-SMA. (**B**) Immunofluorescence section of mouse liver TGF-β. (**C**) Immunofluorescence section of BAX in mouse liver. (**D**) Immunofluorescence section of BCL-2 within mouse liver. (**E**) Immunofluorescence quantification of BAX/BCL-2 within mouse liver. (**F**) Immunofluorescence quantification of α-SMA/TGF-β in mouse liver.

According to our results, the liver fibrosis degree was higher in untreated D-gal group. Therefore, aging induced liver fibrosis. The improvement of liver metabolism by Rg3 may be associated with inhibiting apoptosis while reducing TGF-β [22] and α-SMA secretion [23]. Based on immunofluorescence results, relative to D-gal group, TGF-β and α-SMA secretion in the liver of Rg3 treatment group was alleviated (Figure 4A, 4B). Through determining BAX and BCL-2 protein levels within the liver, we found that BCL-2 (anti-apoptotic protein) expression elevated, while BAX (pro-apoptotic protein) expression markedly declined in Rg3 treatment group (Figure 4C, 4D).

## DISCUSSION

Senility related liver fibrosis is a common clinical disease in the elderly. Several studies have shown that the enzyme activity in the liver changes first under the induction conditions of oxidants and inflammatory factors. Rg3 can down-regulate abnormally elevated AST and ALT, thereby improving liver function. As an important place for lipid metabolism, TC, LDL-C, TG and HDLC contents during aging process are abnormal. However, after taking Rg3, the serum lipid marker content of mice gradually recovered to normal [24, 25]. As a key protein in the regulation of inflammatory response, the expression of NF-KB is positively regulated with TNF-α, IL-6, IL-1β and IL-18 [17]. Rg3 can inhibit ROS generation, inhibit mitochondrial swelling and antagonize cellular inflammation, senescence and apoptosis by extracting rat brain mitochondria *in vitro* and induced by H_2_O_2_. Rg3 can also inhibit IL-6 and IL-18 production, and improve cell senescence [12].

In this study, D-gal induced liver tissue apoptosis, hepatocyte damage and severe fibrosis in mice [26]. Serum levels of TNF-α, IL-6, IL-1β and IL-18 were determined through ELISA. Expectedly, serum secretion of inflammatory cytokines was reduced after Rg3 treatment, and NF-KB protein expression in liver was decreased after Rg3 treatment, as measured by immunofluorescence. In addition, extensive research has shown that inflammatory responses promote apoptosis, which was also confirmed in the current study. After treatment with Rg3, Bcl-2/Bax levels were, with Bcl-2 and Bax showing significant up- and down-regulation, separately.

## CONCLUSIONS

The present experiments showed that ginsenoside Rg3 attenuated D-gal-mediated mouse liver injury. Based on our results, ginsenoside Rg3 is the potential novel drug used to treat senile liver injury.
